# Effects of Different Roughages on Growth Performance, Nutrient Digestibility, Ruminal Fermentation, and Microbial Community in Weaned Holstein Calves

**DOI:** 10.3389/fvets.2022.864320

**Published:** 2022-07-12

**Authors:** Jichao Li, Hongxia Lian, Airong Zheng, Jiangfan Zhang, Pengfei Dai, Yan Niu, Tengyun Gao, Ming Li, Liyang Zhang, Tong Fu

**Affiliations:** ^1^Henan International Joint Laboratory of Nutrition Regulation and Ecological Raising of Domestic Animal, College of Animal Science and Technology, Henan Agricultural University, Zhengzhou, China; ^2^Henan Forage Feeding Technology Extension Station, Zhengzhou, China

**Keywords:** calves, roughages, growth, nutrient digestibility, rumen fermentation, bacterial community

## Abstract

This study aimed to assess the effects of feeding with different forage sources and starter concentrations on growth performance, nutrient digestibility, ruminal fermentation, and the microbial community in weaned Holstein calves. A total of 54 Holstein calves (body weight (BW) = 77.50 ± 5.07 kg; age = 70 ± 2.54 days) were assigned to 1 of 3 treatment groups (*n* = 18/group) that were offered diets with different forages: (1) peanut vine (PV), (2) oat hay (OH), or (3) an alfalfa hay + oat hay combination (alfalfa hay:oat hay =1:1, AO). Starter and forage intakes were recorded daily, while BW and growth parameters were assessed at 15-day intervals. The apparent digestibility of nutrients was determined. Ruminal fluid samples were collected and used to detect relevant indicators. A difference was observed for the forage × age interaction for all feed, nutrient intake, BW, ADG, and body structure parameters (*P* < 0.05). The final BW, average daily feed intake (ADFI), and average daily gain of the PV calves were higher than those of calves from the other groups (*P* < 0.05). The ruminal propionate concentration evidently increased in calves of the AO group (*P* < 0.05). The abundances of *Rikenellaceae_RC9_gut_group* and *Shuttleworthia* showed distinct responses to feeding with different forages (*P* < 0.05) at the genus level. The relative abundance of *Shuttleworthia* was negatively related to rumen pH and acid detergent fiber digestibility (*P* < 0.05) and strongly positively related to propionate concentration (*P* < 0.01). A positive correlation was found between *Ruminococcus_1* abundance and butyrate concentration and neutral detergent fiber digestibility (*P* < 0.05). The relative abundances of *Succiniclasticum* and *Prevotella_7* were negatively related to butyrate concentration (*P* < 0.05). In conclusion, there was an interaction between the factors (forage × age). The peanut vine used as a forage source promoted a higher starter concentrate intake compared to other diets and increased with the calves' age. The growth performance and rumen bacterial community of the calves were further improved. These results indicate that peanut vine can be used as the main source of forage in the diets of weaned calves.

## Introduction

An adequate supply of forage is considered essential for the transition of calves into functional ruminants (1). Adequate forage fed to calves has been found to decrease the incidence of coalescing ruminal papillae, increase muscle thickness, improve the rumen fermentation environment, and alleviate hyperkeratosis and plaque formation in the rumen epithelium (2), promoting rumen development. However, to meet the growing needs of beef cattle and maximize the efficiency of production, it is necessary to feed with high-concentration diets (HCDs) (3). HCDs contain large amounts of carbohydrates that have been found to reduce the rumen buffering capacity through the rapid accumulation of short-chain fatty acids (SCFAs), leading to subacute rumen acidosis (4). An inadequate supply of forage causes short-term metabolic disorders in calves, such as bloat, acidosis, and reduced volatile fatty acid (VFA) absorption (5). The developmental dysplasia of the rumen reduces feed intake and weight gain in calves and results in other negative effects during the growth and production periods. Therefore, it is recommended that sufficient forage be supplied to growing calves (6).

A popular feeding strategy to ensure normal development of the rumen epithelium and improve growth performance is to offer pelleted starter feed as well as forage. Alfalfa hay concurrently fed with pelleted starter feed is beneficial for calf growth (7). Alfalfa hay and starter feed improve the health and growth of yak calves during preweaning (8). Supplementation with fiber carbohydrates, such as alfalfa hay, has been observed to increase the ruminal abundance of Bacteroidetes and improve pH (9). However, feeding with alfalfa hay has been associated with reduced nitrogen utilization, increased incidence of trophic diarrhea, and even increased nitrogen output to the environment because of the high protein content of alfalfa hay (10). Replacing part of the alfalfa hay with oat hay can help overcome the above disadvantages of feeding with alfalfa hay alone (10). Oat hay was found to improve rumen fermentation parameters and help maintain the growth of rumen microorganisms, contributing to the successful transition of calves to functional ruminants (11). Alternatively, providing pelleted starter feed with oat hay was also beneficial (12).

Although it has been suggested that calves require forage to improve performance, behavior, and utilization efficiency (13), the optimal variety of forage has not been clearly defined. The cost of feeding animals has steadily increased due to the rising cost of ingredients (1). Peanut vine, an unconventional type of forage, has received increasing attention. The crude protein (CP) content, which is up to 12%, makes peanut vine extremely valuable for herbivores. Compared to alfalfa hay, it also contains more digestible dry matter (DM), neutral detergent fiber (NDF), and acid detergent fiber (ADF) (14). Another advantage is its high yield. The identification of cheaper forage to replace high-quality forage or of ways to reduce high-quality forage feeding has been of interest in recent years, but the optimal source of forage for weaned calves needs further research.

In view of this, we hypothesized that feeding peanut vine to weaned calves could improve growth performance and the rumen environment. Therefore, a trial for feeding calves with different forage sources was carried out, and the relevant indicators were analyzed to explore better feeding strategies during the early postweaning period.

## Materials and Methods

### Animals, Experimental Design, and Diets

The experiment was conducted at the College of Animal Science and Veterinary Medicine of Henan Agricultural University (Xuchang, China). The feeding trial was carried out according to the protocols proposed by the Institutional Animal Care and Use Committee (IACUC) of Henan Agriculture University (Zhengzhou, China) (Permit Number: Hnnd2019082002).

A total of 54 male Holstein calves (body weight (BW) = 77.50 ± 5.07 kg; age = 70 ± 2.54 days) were blocked according to their BW and age before being randomly allocated into 1 of 3 treatment groups (18 calves per group) that were offered diets with different chopped forages: (1) peanut vine (PV), (2) oat hay (OH), or (3) an alfalfa hay + oat hay combination (alfalfa hay:oat hay =1:1, AO). The calves were required to adapt to the diets within 7 days, followed by a 60-day feeding experiment. The formulation of the concentrate and the nutrient levels in the concentrate and forage are shown in Tables 1, 2. Calves were fed in separate pens in a calf hutch (4.7 m × 1.5 m) and provided starters according to the published estimation equations and values of the Agricultural and Food Research Council (AFRC, 1993) and according to BW (15), with *ad libitum* access to chopped forages and water for the entire trial. The method involved feeding calves with concentrate and forage separately.

**Table 1:** Composition and nutrient levels of the concentrate.

**Items**	**Content**
Ingredients (% of dry matter, unless noted)	
Corn	42.00
Soybean meal	24.00
Wheat bran	15.00
Extrude soybean	4.00
Dried distiller's grains with soluble	11.00
CaHPO_4_	0.50
Limestone	1.50
NaCl	1.00
Premix^(1)^	1.00
Total	100.00
Nutrient levels (% of dry matter, unless noted)	
Dry matter	91.35
Gross energy / (MJ/kg)	15.42
Crude protein	20.69
Neutral detergent fiber	16.93
Acid detergent fiber	5.03
Ether extract	4.31
Ash	7.72
Calcium	1.21
Phosphorus	0.61
NFC^(2)^	50.35

^(1)^*The premix provided the following per kg of the concentrate: VA 15 000 IU, VD 5 000 IU, VE 50 mg, Fe 90 mg, Cu 12.5 mg, Mn 60 mg, Zn 100 mg, Se 0.3 mg, I 1.0 mg, Co 0.5 mg. ^(2)^NFC (%) =100–(NDF + CP + EE + ash), NFC: non-fibrous carbohydrate*.

**Table 2:** Nutrient levels of forages.

	**Forages**		
**Items**	**Peanut vine**	**Oat hay**	**Alfalfa hay**
Nutrient levels (% of dry matter,			
unless noted)			
Dry matter	91.95	92.72	91.76
Gross energy / (MJ/kg)	13.97	14.21	14.90
Crude protein	8.95	9.02	15.72
Neutral detergent fiber	39.34	62.78	39.05
Acid detergent fiber	31.96	38.10	27.78
Ether extract	2.17	2.28	2.13
Ash	11.03	9.17	10.93
Calcium	1.27	0.35	1.71
Phosphorus	0.14	0.28	0.24
NFC^(1)^	38.51	16.75	32.17

^(1)^*NFC (%) =100 – (NDF + CP + EE + ash); NFC, non-fibrous carbohydrate*.

### Measurements and Sample Collection

The average daily feed intake (ADFI) was further calculated by recording the amount of feed provided and the amount not consumed each day. Body structure traits such as BW, body length, body height, and chest girth of the calves were measured at 15-day intervals. Measurements were recorded on 2 consecutive days. The average daily gain (ADG) and feed efficiency [kg of total dry matter intake (DMI)/kg of BW gain] were calculated from these measurements. The eight calves in each group with similar body weight were selected for a 3-day adaptation period and a 4-day digestibility trial by total fecal and urine collection after day 60 of the experiment. The feces collected over 24 h were weighed and mixed evenly and then immediately stored with sulfuric acid. Feed samples were collected and stored at −20°C for analysis as previously described (16).

Ruminal fluid samples of nine calves per experimental group were collected on day 60 using an oral stomach tube after 2 h of feeding. The pH was measured using a calibrated pH meter (HI 8318, Hanna Instruments, Cluj-Napoca, Romania). The remaining samples were separated into three tubes for further analysis of ammonia nitrogen (NH_3_-N), VFA, and rumen microorganisms.

### Chemical Analysis

The samples were analyzed for DM (method 934.01), CP (method 988.05), ether extract (EE) (method 920.39), ash (method 942.05), and calcium (Ca) and phosphorus (P) (method 945.46) by AOAC (2012) (17). NDF and ADF levels were determined with sodium sulfide and heat-stable α-amylase by using an ANKOM fiber analyzer (Fiber Analyzer A200; Ankom Technology, NY, USA) (18). The gross energy (GE) levels were analyzed with an oxygen bomb calorimeter (ZDHW-8000, Huano Electronic Technology Co., Ltd., Hebi, China). The NH_3_-N concentration was analyzed by phenol hypochlorite colorimetry (19). The VFA concentration was determined by ion chromatography (ICS-3000, Dionex Corporation, California, USA).

### Ruminal Bacterial Community Composition Analysis

Ruminal fluid samples were sent to Majorbio BioPharm Technology Co., Ltd., (Shanghai, China) for bacterial community composition analysis.

#### DNA Extraction and PCR Amplification

Genomic DNA of the microbial community was extracted using the E.Z.N.A.^®^ Soil DNA Kit (Omega Biotek, Norcross, GA, USA). The hypervariable region V3–V4 of the bacterial 16S rRNA gene was amplified with the primer pair 338F (5'-ACTCCTACGGGAGGCAGCAG-3') and 806R (5'-GGACTACHVGGGTWTCTAAT-3') by an ABI GeneAmp^®^ 9700 PCR thermocycler (ABI, CA, USA). PCR amplification of the 16S rRNA gene was performed as follows: initial denaturation at 95°C for 3 min, followed by 27 cycles of denaturing at 95°C for 30 s, annealing at 55°C for 30 s, and extension at 72°C for 45 s, a single extension at 72°C for 10 min, and a final extension at 10°C. Three replicate PCRs were performed. The PCR product was extracted, purified, and quantified using a Quantus™ Fluorometer (Promega, USA).

#### Illumina MiSeq Sequencing

Paired-end sequencing was performed on an Illumina MiSeq PE 300/NovaSeq PE250 platform (Illumina, San Diego, USA). The raw reads were deposited in the NCBI Sequence Read Archive (SRA) (Accession Number: PRJNA786062).

#### Processing of Sequencing Data

Fastp version 0.20.0 and FLASH version 1.2.7 were used for quality filtering and merging in the process of demultiplexing the raw 16S rRNA gene sequencing reads. The operational taxonomic units (OTUs) were clustered using UPARSE version 7.1. Principal coordinate analysis (PCoA) and correlation heatmap analysis were performed with R (version 3.3.1).

### Statistical Analysis

The data were analyzed using SAS/STAT software (version 9.4, SAS Institute Inc., Cary, NC). Feed intake and growth data were repeatedly measured using a mixed model that included fixed effects of forage, age, and the forage × age interaction and the random effects of the block and calves within the block. A total of 5 blocks were created. One-way ANOVA was used to analyze the fermentation parameters of the rumen and bacterial diversity. Statistically significant differences among groups were evaluated by Duncan's test. Differences were declared to be statistically significant at *P* < 0.05. Least squares means with the standard error of the means are reported.

## Results

### Feed Intake and Growth Performance

There was an interaction between forage and age. The ADFI, starter intake, and forage intake of the calves in the PV group were higher than those of the calves in the OH group and AO group (*P* < 0.05) (Table 3). The CP intake of calves in the PV and AO groups was higher than that in the OH group (*P* < 0.05), the EE intake of calves in the PV group was higher than that in the OH and AO groups (*P* < 0.05), and the NDF intake of calves in the OH and AO groups was higher than that in the PV group (*P* < 0.05) (Table 3). In addition, a difference was noted for age for all feed and nutrient intake (*P* < 0.01), and a difference was observed for the forage × age interaction for all feed and nutrient intake (*P* < 0.05). The ADG of calves in the PV group and AO group was higher than that of the calves in the OH group (*P* < 0.01). The body weight and chest girth of calves in the PV group were greater than those of calves in the OH group and AO group (*P* < 0.01) (Table 4). The BW, ADG, and body structure measurements were significantly affected by the forage, calf age, or the interaction of forage and age (*P* < 0.05) (Table 4).

**Table 3:** Least squares means of feed intake for calves fed with different diets.

	**Forage^1^**				**Age**						***P*** **–value^2^**		
**Items**	**PV**	**OH**	**AO**	**SEM**	**1**	**2**	**3**	**4**	**5**	**SEM**	**Forage**	**Age**	**Forage × Age**
Average daily feed intake, DM, kg/d	4.48^a^	3.49^c^	3.99^b^	0.07	3.25^d^	3.26^d^	4.01^c^	4.51^b^	4.92^a^	0.06	<0.001	<0.001	0.002
Starter intake, DM, kg/d	1.90^a^	1.79^c^	1.85^b^	0.02	1.50^d^	1.50^d^	1.99^c^	2.07^b^	2.16^a^	0.01	0.001	<0.001	<0.001
Forage intake, DM, kg/d	2.59^a^	1.70^c^	2.15^b^	0.07	1.75^d^	1.76^d^	2.02^c^	2.44^b^	2.77^a^	0.06	<0.001	<0.001	0.004
CP intake, kg/d	1.33^a^	1.04^b^	1.32^a^	0.02	1.00^d^	1.00^d^	1.24^c^	1.39^b^	1.52^a^	0.02	<0.001	<0.001	<0.001
EE intake, kg/d	0.29^a^	0.23^c^	0.26^b^	<0.01	0.21^d^	0.21^d^	0.26^c^	0.30^b^	0.32^a^	<0.01	<0.001	<0.001	<0.001
NDF intake, kg/d	2.52^b^	2.78^a^	2.71^a^	0.04	2.16^d^	2.19^d^	2.69^c^	3.02^b^	3.30^a^	0.04	<0.001	<0.001	<0.001
ADF intake, kg/d	1.66^a^	1.51^b^	1.52^b^	0.03	1.27^d^	1.27^d^	1.57^c^	1.77^b^	1.93^a^	0.02	<0.001	<0.001	0.003

a−d *Means within a row with different superscripts differ (P < 0.05)*.

1 *PV, the peanut vine used as forage source; OH, the oat hay used as forage source; AO, alfalfa hay + oat hay combination used as forage source (alfalfa hay:oat hay = 1:1)*.

2 *The feed intake data were repeatedly measured using a mixed model that included fixed effects of forage, age, and forage × age interaction, the random effects of the block, and calf within the block*.

**Table 4:** Least squares means of ADG (kg/day) and body structure measurements (cm) for calves fed with different diets.

	**Forage^>1^**				**Age**						***P*** **-value^2^**		
**Items**	**PV**	**OH**	**AO**	**SEM**	**1**	**2**	**3**	**4**	**5**	**SEM**	**Forage**	**Age**	**Forage × Age**
Average daily gain, kg/d	0.82^a^	0.57^b^	0.76^a^	0.02	–	–	–	–	–	–	<0.001	<0.001	0.001
Body weight, kg	103.07^a^	95.38^b^	98.72^b^	1.32	77.66^e^	91.46^d^	96.54^c^	106.29^b^	123.33^a^	0.80	<0.001	<0.001	<0.001
Body height, cm	95.28^a^	94.21^ab^	93.74^b^	0.50	88.62^e^	92.16^d^	93.43^c^	96.34^b^	101.49^a^	0.38	0.090	<0.001	0.006
Body length, cm	97.98^b^	97.73^b^	100.11^a^	0.46	89.27^e^	94.90^d^	99.82^c^	101.15^b^	107.89^a^	0.50	<0.001	<0.001	<0.001
Chest girth, cm	108.87^a^	105.94^b^	107.23^b^	0.56	99.40^e^	105.47^d^	107.50^c^	108.58^b^	115.79^a^	0.47	0.002	<0.001	0.002

a−e *Means within a row with different superscripts differ (P < 0.05)*.

1 *PV, the peanut vine used as forage source; OH, the oat hay used as forage source; AO, alfalfa hay + oat hay combination used as forage source (alfalfa hay:oat hay = 1:1)*.

2 *The growth data were repeatedly measured using a mixed model that included fixed effects of forage, age, and forage × age interaction, the random effects of block, and calf within block*.

### Apparent Nutrient Digestibility

The apparent digestibility of DM in calves in the PV group was higher than that in calves in the OH group (*P* < 0.05) (Table 5). The apparent digestibility of organic matter (OM) and Ca in calves in the PV group was higher than that in calves in the OH group and AO group (*P* < 0.05). There was no significant difference in the apparent digestibility of the other components (*P* > 0.05) (Table 5).

**Table 5:** Effects of diets on apparent nutrient digestibility in calves.

	**Groups^1^**				
**Items**	**PV**	**OH**	**AO**	**SEM**	***P*-value**
Dry matter, %	75.00^a^	67.25^b^	71.36^ab^	0.07	0.022
Organic matter, %	78.13^a^	72.83^b^	74.75^b^	0.11	0.004
Crude protein, %	67.75	69.00	72.00	0.05	0.277
Ether extract, %	76.75	75.38	77.87	0.05	0.575
Neutral detergent fiber, %	50.57	54.14	52.43	0.08	0.353
Acid detergent fiber, %	42.53	46.32	44.72	0.10	0.129
Calcium, %	64.13^a^	50.88^b^	62.00^a^	0.09	0.002
Phosphorus, %	81.00	82.88	84.25	0.05	0.463

a−b *Means within a row with different superscripts differ (P < 0.05)*.

1 *PV, the peanut vine used as forage source; OH, the oat hay used as forage source; AO, alfalfa hay + oat hay combination used as forage source (alfalfa hay:oat hay =1:1)*.

### Rumen Fermentation Parameters

The ruminal pH of calves in the PV group and OH group was relatively higher (*P* < 0.05) (Table 6). In addition, the propionate concentration of the calves in the PV group and AO group was evidently increased (*P* < 0.05). The butyrate concentration was higher in the rumen fluids of calves in the PV group, but the difference was not significant (*P* > 0.05) (Table 6).

**Table 6:** Effects of different forages on rumen fermentation parameters of Holstein male calves.

	**Groups^1^**				
**Items**	**PV**	**OH**	**AO**	**SEM**	***P*-value**
pH	6.56^a^	6.65^a^	6.22^b^	0.07	0.023
Ammonia nitrogen (mg/dL)	12.06	11.53	14.83	1.38	0.176
Total volatile fatty acid (mmol/L)	58.47	50.74	60.36	2.44	0.241
Acetate (mmol/L)	31.18	28.29	34.06	1.32	0.209
Propionate (mmol/L)	16.35^a^	13.22^b^	17.43^a^	0.85	0.044
Butyrate (mmol/L)	10.94	9.23	8.88	0.54	0.262
Acetate/propionate	1.99	2.15	1.99	0.06	0.364

a−b *Means within a row with different superscripts differ (P < 0.05)*.

1 *PV, the peanut vine used as forage source; OH, the oat hay used as forage source; AO, alfalfa hay + oat hay combination used as forage source (alfalfa hay:oat hay =1:1)*.

### Microbiota Diversity and Composition

A total of 1,614,585 bacterial sequences were obtained. The average length of all fragments after quality checks and filtering was 420 bp. No difference in the alpha diversity index was found (*P* > 0.05) (Table 7). These sequences were clustered into 1,489 OTUs, representing 24 phyla. The eight predominant phyla were *Bacteroidetes* (64.59%), *Firmicutes* (29.87%), *Proteobacteria* (1.83%), *Actinobacteria* (1.27%), *Tenericutes* (0.79%), *Spirochaetes* (0.79%), *Patescibacteria* (0.38%), and *Kiritimatiellaeota* (0.09%; Table 8). Overall, the relative abundances of *Bacteroidetes* and *Firmicutes* were higher at the phylum level. There were significant differences in the abundances of other bacteria at the phylum level among the groups (*P* < 0.01).

**Table 7:** Effect of different forages on alpha diversity indexes in the rumen bacterial community.

	**Groups^1^**				
**Items**	**PV**	**OH**	**AO**	**SEM**	***P*-value**
Reads	57, 672.67	59, 353.78	62, 371.89	1, 301.81	0.341
Sobs	526.89	505.78	525.22	13.53	0.791
Shannon	3.54	3.26	3.56	0.11	0.457
Simpson	0.09	0.13	0.08	0.01	0.225
Ace	633.30	646.95	657.29	13.85	0.791
Chao1	643.88	640.67	658.95	14.61	0.871

1 *PV, the peanut vine used as forage source; OH, the oat hay used as forage source; AO, alfalfa hay + oat hay combination used as forage source (alfalfa hay:oat hay = 1:1)*.

**Table 8:** Effect of different forages on phylum-level diversity (% of total sequences) in the rumen bacterial community.

	**Groups^1^**				
**Items**	**PV**	**OH**	**AO**	**SEM**	***P*-value**
Bacteroidetes	62.10	71.00	60.66	2.66	0.235
Firmicutes	32.55	23.75	33.30	2.43	0.210
Proteobacteria	1.37	2.43	1.70	0.36	0.485
Actinobacteria	1.13	1.08	1.61	0.21	0.541
Tenericutes	1.12	0.51	0.75	0.13	0.197
Spirochaetes	1.31	0.42	0.63	0.18	0.113
Patescibacteria	0.12	0.32	0.71	0.16	0.303
Kiritimatiellaeota	0.06	0.20	0.00	0.06	0.497
Others	0.24^b^	0.29^b^	0.63^a^	0.05	0.007

a−b *Means within a row with different superscripts differ (P < 0.05)*.

1 *PV, the peanut vine used as forage source; OH, the oat hay used as forage source; AO, alfalfa hay + oat hay combination used as forage source (alfalfa hay:oat hay = 1:1)*.

**Figure 1:**
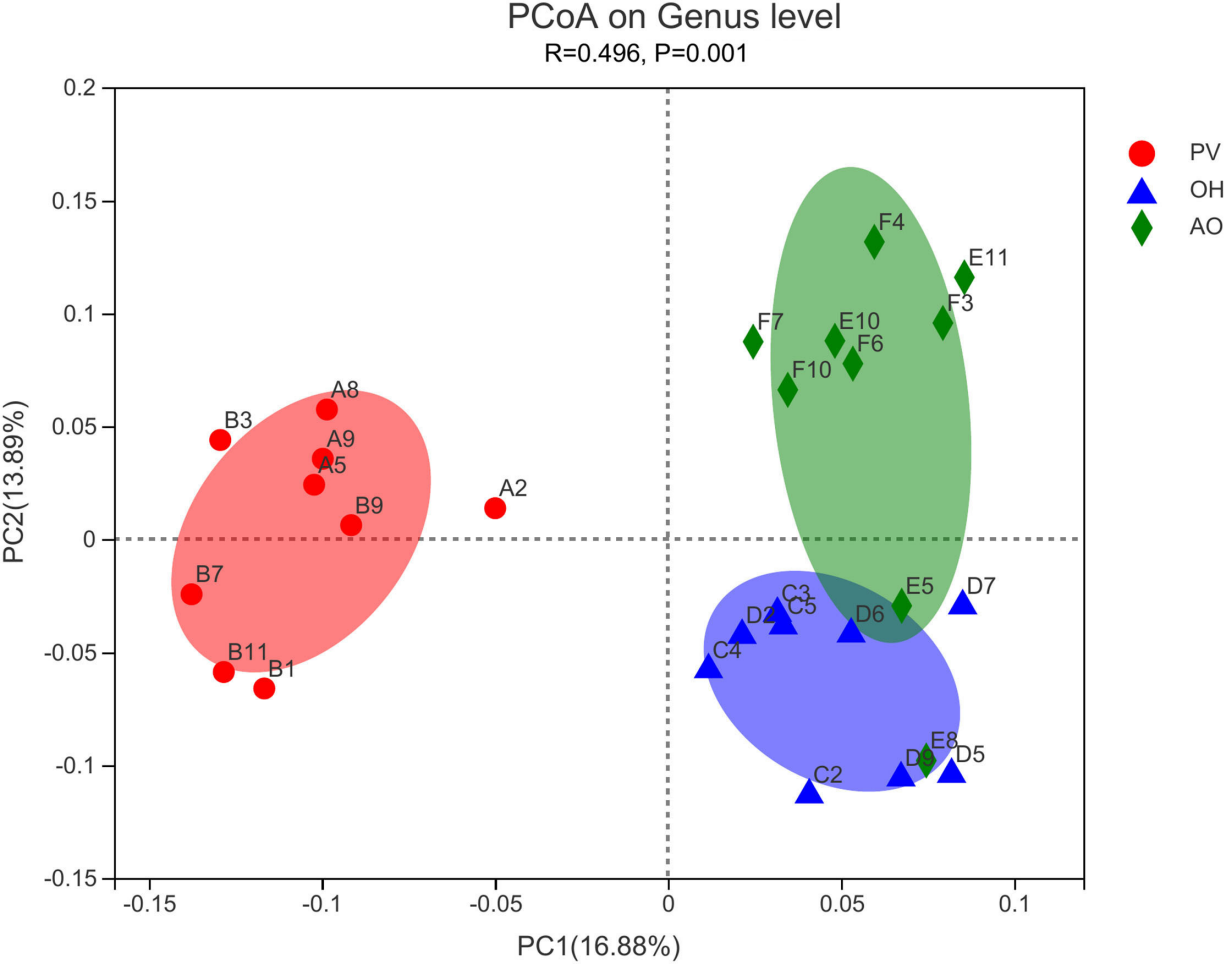
Principal coordinate analysis (PCoA) showed that the community composition of different samples could reflect the differences and distances. PV, peanut vine used as a forage source; OH, oat hay used as a forage source; AO, the alfalfa hay + oat hay combination used as a forage source (alfalfa hay:oat hay = 1:1).

A total of 333 bacterial genera were detected in the samples. The predominant taxa were *Prevotella-1* (34.76%), *Rikenellaceae_RC9_gut_group* (15.97%), *Erysipelotrichaceae_UCG-002* (6.54%), *Christensenellaceae_R-7_group* (3.24%), and *Prevotella_7* (2.88%; Table 9). The relative abundance of *Rikenellaceae_RC9_gut_group* in calves in the OH group was higher than that in the PV group (*P* < 0.05). The relative abundance of *unclassified_o__Bacteroidales* in the PV group was higher than that in the other groups (*P* < 0.01). The relative abundance of *Shuttleworthia* in the PV group was higher than that in the OH group (*P* < 0.05). There was a clear separation of clusters on the PCoA plot among the PV group, the OH group, and the AO group (Figure 1). PC1 and PC2 accounted for 13.89% and 16.88% of the total variance, respectively. An ANOSIM showed no difference among groups (*P* = 0.001).

**Table 9:** Effect of different forages on genus-level diversity (% of total sequences) in the rumen bacterial community.

		**Groups^1^**				
**Phylum**	**Genus**	**PV**	**OH**	**AO**	**SEM**	***P*-value**
Bacteroidetes	*Prevotella_1*	28.43	43.63	32.23	4.31	0.339
	*Rikenellaceae_RC9_gut_group*	4.36^b^	14.32^a^	10.56^ab^	1.54	0.021
	*Prevotella_7*	0.93	3.03	4.69	1.05	0.357
	*norank_f__F082*	2.92	2.27	2.25	1.03	0.959
	*Prevotellaceae_Ga6A1_group*	1.78	0.35	2.69	0.92	0.602
	*norank_f__p-2534-18B5_gut_group*	1.38	1.46	1.11	0.28	0.879
	*Prevotellaceae_UCG-001*	1.16	0.87	0.88	0.17	0.752
	*Bacteroidales_UCG-001*	0.00	2.03	0.41	0.30	0.009
	*Alloprevotella*	0.92	0.48	0.57	0.11	0.241
	*unclassified_f__Prevotellaceae*	0.31	0.42	0.70	0.09	0.230
	*Prevotellaceae_UCG-003*	0.57	0.77	0.12	0.14	0.175
	*U29-B03*	0.24	0.41	0.46	0.15	0.823
	*Prevotella_9*	0.08	0.27	0.47	0.09	0.260
	*unclassified_o__Bacteroidales*	0.55^a^	0.04^b^	0.03^b^	0.08	0.007
	*norank_f__Bacteroidales_RF16_group*	0.36	0.04	0.05	0.06	0.054
Firmicutes	*Erysipelotrichaceae_UCG-002*	6.93	3.00	9.70	1.60	0.238
	*Christensenellaceae_R-7_group*	3.36	3.07	3.30	0.79	0.988
	*Ruminococcaceae_UCG-014*	1.44	2.29	2.13	0.35	0.586
	*Lachnospiraceae_NK3A20_group*	1.49	2.06	1.51	0.27	0.634
	*Succiniclasticum*	1.09	1.34	1.75	0.29	0.656
	*Ruminococcus_1*	1.51	0.48	0.91	0.43	0.634
	*Ruminococcaceae_NK4A214_group*	0.84	0.71	0.95	0.09	0.565
	*Sharpea*	0.67	0.20	1.22	0.22	0.152
	*Shuttleworthia*	1.22^a^	0.07^b^	0.72^ab^	0.18	0.028
	*Ruminococcus_2*	0.25	1.31	0.41	0.22	0.118
	*Ruminococcaceae_UCG-013*	0.76	0.28	0.56	0.12	0.279
	*Ruminococcaceae_UCG-002*	0.34	0.74	0.48	0.10	0.294
	*Lachnospiraceae_NK4A136_group*	0.68	0.67	0.13	0.14	0.201
	*[Eubacterium]_coprostanoligenes_group*	0.57	0.41	0.41	0.05	0.401
	*[Eubacterium]_ruminantium_group*	0.35	0.65	0.30	0.09	0.240
	*Pseudobutyrivibrio*	0.34	0.43	0.49	0.11	0.875
	*[Ruminococcus]_gauvreauii_group*	0.57	0.47	0.25	0.15	0.683
Proteobacteria	*Succinivibrio*	0.72	0.86	0.34	0.24	0.681
	*Succinivibrionaceae_UCG-001*	0.09	0.94	0.55	0.24	0.369
Actinobacteria	*Olsenella*	0.62	0.80	1.27	0.19	0.372
Others		14.74	8.59	12.65	1.24	0.120

a−b *Means within a row with different superscripts differ (P < 0.05)*.

1 *PV, the peanut vine used as forage source; OH, the oat hay used as forage source; AO, alfalfa hay + oat hay combination used as forage source (alfalfa hay:oat hay = 1:1)*.

### Correlations Between Environmental Factors and Microbiota Structure

**Figure 2:**
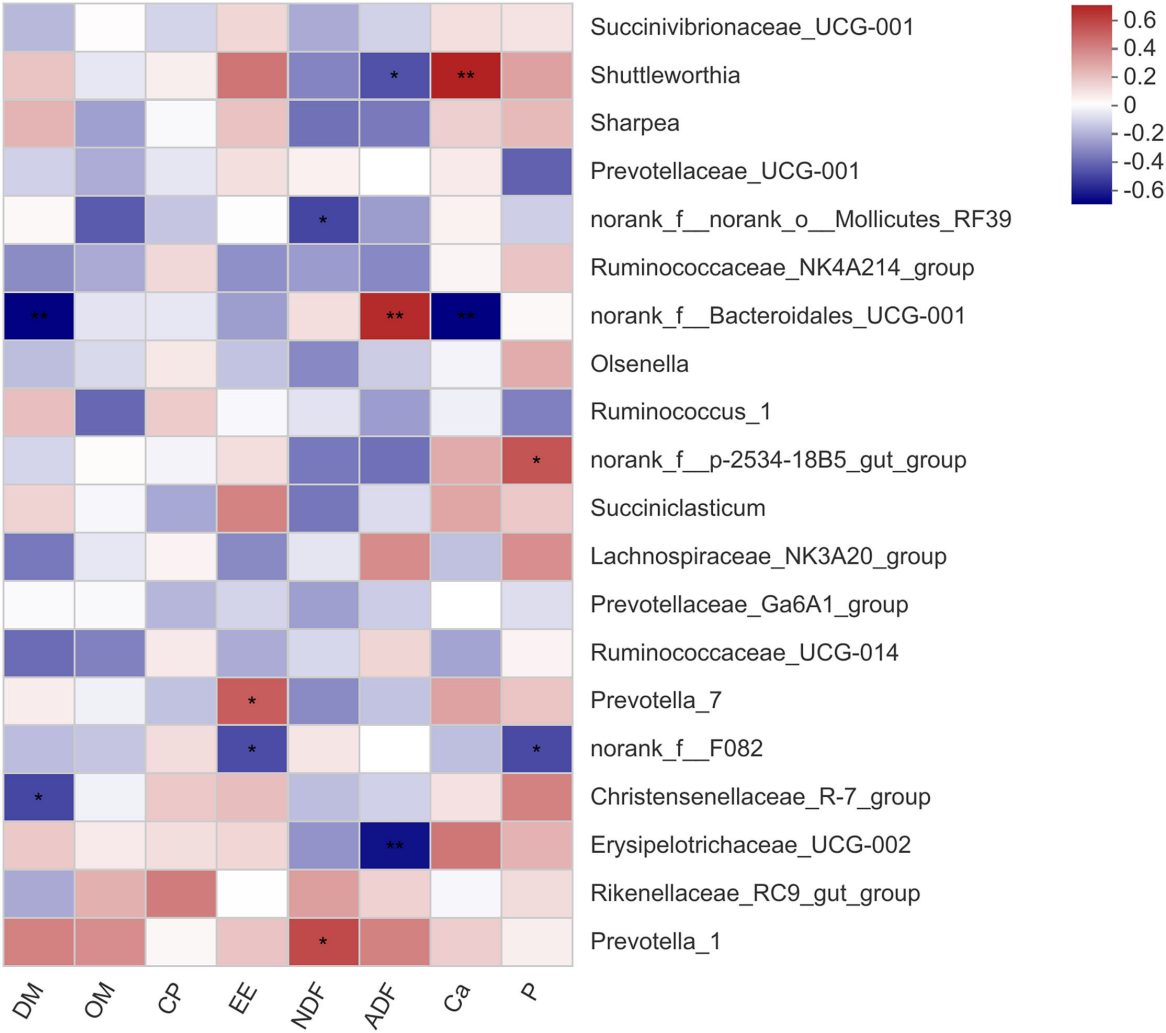
Heatmaps showing the correlations between the apparent digestibility of nutrients and the relative abundance of bacterial genera. DM, dry matter; OM, organic matter; CP, crude protein; EE, ether extract; NDF, neutral detergent fiber; ADF, acid detergent fiber; Ca, calcium; P, phosphorus. * 0.01 < *P* ≤ 0.05, ** 0.001 < *P* ≤ 0.01, *** *P* ≤ 0.001.

**Figure 3:**
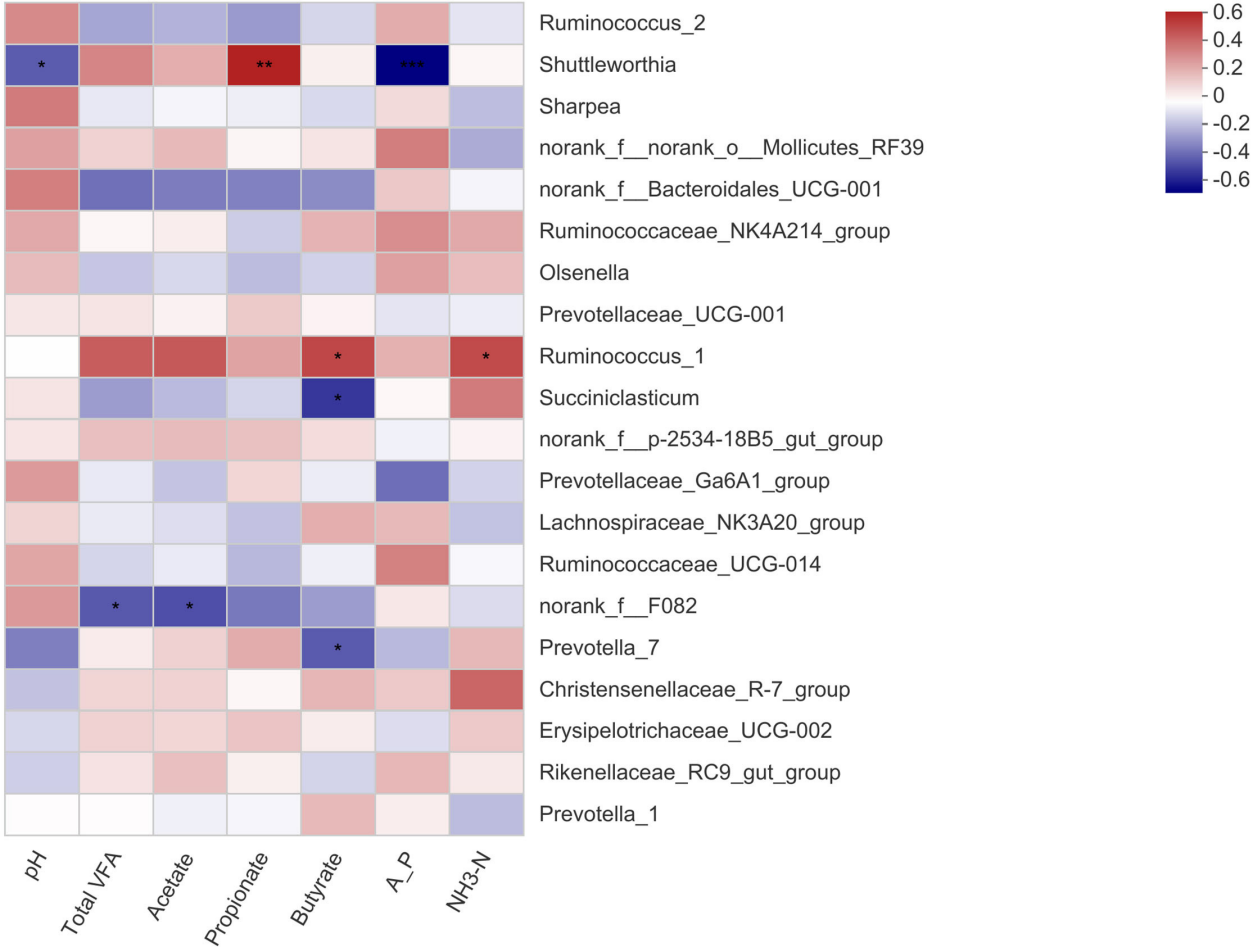
Heatmaps showing the correlations between fermentation parameters of the rumen and the relative abundance of bacterial genera. pH, hydrogen ion concentration; Total VFA, total volatile fatty acid; A_P, acetate/propionate; NH_3_-N, ammonia nitrogen. * 0.01 < *P* ≤ 0.05, ** 0.001 < *P* ≤ 0.01, *** *P* ≤ 0.001.

The correlation between the microbial genera and the apparent digestibility of nutrients in the calves is shown in Figure 2. The relative abundance of *Shuttleworthia* was negatively related to ADF digestibility (*P* < 0.05) and strongly positively related to Ca digestibility (*P* < 0.01). The *norank_f__Bacteroidales_UCG-001* abundance was strongly negatively related to DM digestibility (*P* < 0.01) and positively related to ADF digestibility (*P* < 0.01). There was a positive correlation between the relative abundance of *Prevotella_1* and NDF digestibility (*P* < 0.05). The correlation between the microbial genera and rumen fermentation parameters in the calves is shown in Figure 3. The relative abundance of *Shuttleworthia* was negatively related to rumen pH (*P* < 0.05) and positively related to propionate concentration (*P* < 0.05). The relative abundance of *Succiniclasticum* was negatively related to the butyrate concentration (*P* < 0.05).

## Discussion

All feed and nutrient intake parameters were significantly affected by forage, calf age, or the interaction between forage and age. The source of dietary NDF has been found to affect the growth performance of calves (5). Low-quality forages are abundant in NDF, which induces satiety (20); thus, the physical fill can reduce feed intake. This may explain why the intake of calves in the OH group was lower. It is well known that the amount of NDF affects the feed intake and growth performance of calves regardless of their source. According to this study, with the combination of oat hay and alfalfa hay, the NDF content was higher, which might have accounted for differences in the ADFI and growth performance among the groups, decreased levels of plaque formation, and increased rumen wall thicknesses. (21). Calves fed with peanut vine had a higher ADG than calves fed with the oat hay and alfalfa: oat hay diets, which is consistent with the previously reported relationship between calf feed intake and ADG (22). Moreover, calves in the PV group had a higher ADG, which was consistent with the ADFI (23). The nutrient intake on the 15th day of the trial did not show a large difference, but nutrient intake increased significantly with age from 15 to 60 days. The calves might have started to exhibit higher feed intake at 85 days of age under dietary induction, accompanied by rapid development with increasing age. The main reason for differences in nutrient intake is the protein and energy levels of the diet itself. The increase in concentrate intake could lead to increased production of VFAs (5), such as butyrate, which could stimulate rumen development. However, the increase in forage intake could improve the rumen pH, decrease the incidence of coalescing ruminal papillae, increase muscle thickness, and improve the rumen fermentation environment (2). The intake of concentrate and forage showed a consistent growth trend, and there were complementary effects, but their interaction effect could not be quantified in this experiment.

Similar to the results for intake, a difference was noted for forage and age in terms of body structure measurements. In summary, the nutrient intake, BW, ADG, and body structure measurements were affected by the forage, calf age, or the interaction between forage and age. Based on this study, the apparent digestibility of DM and OM was affected by feeding with different diets. The underlying reason may be that the forage intake of calves in the PV group was the highest (24). The higher apparent digestibility of OM may have been caused by a greater intake of forages, longer rumination time, and a slower passage rate through the gastrointestinal tract (25). According to the results, the ADG and ADFI of calves in the PV group were higher than those of calves in the other two groups, which was mainly caused by the increased concentrate intake. The digestibility of nutrients (NDF, ADF, and CP) was the same in all the groups, possibly because the different feed intake of each group compensated for the difference in the nutrient content of the diet. The NFC content of peanut vine is relatively higher, which may also be the main reason for the high OM digestibility of calves.

The difference in feed intake among groups resulted in different rumen fermentation results. As calf age increased, voluntary feed intake also increased (19). Calves with increased intake of peanut vine had improved ruminal pH, and oat hay contained a higher level of NDF. Although the intake of calves in the OH group was not as high as that of calves in the PV group, the same effect was produced, which has been found to prevent the adverse effects of low rumen pH (26). The ruminal pH of other groups of calves was also within the normal range. The calves of the PV group and AO group had increased propionate concentrations in the rumen. Propionate is an important glucose precursor (27). In contrast, increased starter intake by calves also increased the propionate and butyrate concentrations and changed the VFA composition, which normally adjusts the rumen fermentation pattern. Butyrate, produced by concentrate fermentation, is vital to promoting the development of the rumen, and its effect is stronger than that of forage (28). According to a previous study, alfalfa hay, oat hay, and rice straw failed to affect the total VFA concentration (29), which was consistent with our results.

The rumen passage rate determined from the DMI of calves was significantly related to NH_3_-N production (30). In addition, the rumen degradable protein content and microbial nitrogen uptake were determined to measure the production of NH_3_-N (31). This study indicated that ruminal NH_3_-N concentrations might not be affected by different forage sources. The PV and AO groups had higher propionate concentrations, higher daily gains, and better feed efficiency than the OH group. However, those treatments also showed higher concentrate intake, which was one of the main factors influencing ruminal propionate concentration, and this was an important factor to consider with these results.

The diversity and richness of the microbial community could make rumen function different (32). At the phylum level, the dominant phyla were Firmicutes and Bacteroidetes. A previous study showed that members of the Bacteroidetes phylum are mainly responsible for protein hydrolysis and carbohydrate degradation, whereas those belonging to the phylum Firmicutes play an important role in energy utilization (33, 34) to meet the high energy requirements of calves during rapid development. The Firmicutes to Bacteroidetes ratio is often used to assess host weight gain because this ratio is linked to energy metabolism (35). This study identified that feeding with different diets might be more suitable for the survival of the phylum Bacteroidetes. The calves fed a diet with peanut vine had a reduced Firmicutes to Bacteroidetes ratio compared with those fed a diet with oat hay, which could be beneficial to the calves.

Importantly, at the genus level, early studies showed that the *Shuttleworthia* genus was highly positively associated with propionate and butyrate (36). In contrast, *Shuttleworthia* species can digest large amounts of hay, which is closely related to fiber digestion (37). In our study, this genus was negatively related to ADF digestibility. In recent years, it has been found that members of *Shuttleworthia* are among the rumen chyme adhesion bacteria in dairy cows and beef cattle and are mainly engaged in the utilization of starch and sugar (38), which is consistent with the positive correlation between the abundance of *Shuttleworthia* and propionate concentration in our results. Ran T et al. (2021) showed a negative correlation between *Shuttleworthia* and lactate and NH_3_-N concentrations (39), which was not observed in this experiment. The abundance of *Shuttleworthia* was closely related to rumen propionate concentration, making it possible to improve rumen function. However, research on this genus is limited, and additional roles in the rumen remain to be further explored.

The relative abundance of *Ruminococcaceae* in the rumen of beef cattle was positively related to feeding efficiency (40). The results demonstrated that the calves in the PV group had a higher utilization capacity for crude fiber, enabling the host to obtain more energy. *Ruminococcus* abundance was also positively related to rumen butyrate concentration. The abundance of the bacteria in the PV group was higher than that in the other groups, indicating that the bacteria might affect the production and utilization of butyrate and thus have an effect on the development and growth of calves. NH_3_-N was the only nitrogen source for the growth of the *Ruminococcus* genus (41), which could explain the positive correlation between *Ruminococcus_1* and NH_3_-N. The genus *Succiniclasticum* has been widely described in rumen communities and is involved in the conversion of succinate to propionate (42). Furthermore, the abundance of propionate-producing bacteria is positively related to animal feed efficiency since propionate is the main precursor of glucogenesis (43). This bacterium was negatively related to butyrate concentration in this study. The abundance of this bacterium in the rumen of the calves in the PV group was relatively low, which meant that butyrate production improved, thus promoting rumen development and improving the ADG.

The *Prevotellaceae* family is a microbial population related to rumen metabolism and genetic diversity, and its main function is to degrade lignocellulosic feed (44). The *Prevotellaceae* family plays a critical role in the metabolism of pectin and protein (45). In this study, feeding different diets to calves changed the abundance of *Prevotella_7*. *Prevotella* usually utilizes a variety of substrates and is considered to be a major producer of propionate (46). However, the data also showed a negative relationship between *Prevotella_7* and butyrate, suggesting that its function in the rumen community needs further study. One of the most dominant bacteria in the rumen of ruminants is *Prevotella_1* (47). The digestibility of NDF also increased with the relative abundance of *Prevotella_1*. The same bacterial population characteristics were also observed in ruminants fed with forage-based diets. In contrast, the presence of the *Rikenellaceae RC9 gut group* has been widely observed in the gastrointestinal tract of several ruminants (48, 49). The decrease in the abundance of the *Rikenellaceae_RC9_gut_group* could improve the anti-inflammatory activity in the intestinal tract (50). *Rikenellaceae_RC9_gut_group* is negatively correlated with glucose metabolism parameters (51). The decrease in *Rikenellaceae_RC9_gut_group* abundance in the PV group might be related to the increase in glucose production, thus increasing daily gain and improving diarrhea in calves. The *Rikenellaceae_RC9_gut_group* abundance was positively correlated with ADG for cattle (42). However, the genus *Rikenellaceae RC9 gut group* exhibited significant negative associations with ADG (52). Therefore, the function of the *Rikenellaceae RC9 gut group* needs to be further explored. These bacteria are closely related to rumen function and improve the rumen fermentation and growth performance of weaned calves.

## Conclusion

Diets containing peanut vine exhibited similar feeding value compared to those using oat and alfalfa hay but increased the feed intake of calves, improved the apparent nutrient digestibility, and changed the rumen fermentation patterns. The screened genera *Shuttleworthia, Ruminococcaceae, Prevotella, Succiniclasticum*, and *Rikenellaceae_RC9_gut_group* can be used as key target bacteria to regulate the rumen function of weaned calves. In conclusion, peanut vine can be used as the main source of forage in feeding strategies for weaned calves.

## Data Availability Statement

The datasets presented in this study can be found in online repositories. The names of the repository/repositories and accession number(s) can be found below: https://www.ncbi.nlm.nih.gov/, PRJNA786062.

## Ethics Statement

The animal study was reviewed and approved by Institutional Animal Care and Use Committee (IACUC) of Henan Agriculture University.

## Author Contributions

Conceptualization: TF and JL. Methodology, data curation, and visualization: HL. Software and supervision: LZ. Validation: ML and TG. Formal analysis and writing—original draft preparation: JL. Investigation: JZ and PD. Resources: AZ and YN. Writing—review and editing: HL and LZ. Project administration: TF. Funding acquisition: TF and TG. All authors contributed to the article and approved the submitted version.

## Funding

This research was funded by the Cooperation Project of Science and Technology of Henan Province, China, Research on energy needs of and starter development of early weaning beef calves (Z152106000029), Henan Agricultural Production Development Fund Project: Research and promotion of peanut vine harvesting, processing, storage, and application technology (2018-15), and supported by the China Agriculture Research System of MOF and MARA.

## Conflict of Interest

The authors declare that the research was conducted in the absence of any commercial or financial relationships that could be construed as a potential conflict of interest.

## Publisher's Note

All claims expressed in this article are solely those of the authors and do not necessarily represent those of their affiliated organizations, or those of the publisher, the editors and the reviewers. Any product that may be evaluated in this article, or claim that may be made by its manufacturer, is not guaranteed or endorsed by the publisher.
